# Diagonally Implicit Symplectic Runge-Kutta Methods with High Algebraic and Dispersion Order

**DOI:** 10.1155/2014/147801

**Published:** 2014-04-01

**Authors:** Y. H. Cong, C. X. Jiang

**Affiliations:** Department of Mathematics, Shanghai Normal University, Shanghai 200234, China

## Abstract

The numerical integration of Hamiltonian systems with oscillating solutions is considered in this paper. A diagonally implicit symplectic nine-stages Runge-Kutta method with algebraic order 6 and dispersion order 8 is presented. Numerical experiments with some Hamiltonian oscillatory problems are presented to show the proposed method is as competitive as the existing same type Runge-Kutta methods.

## 1. Introduction

In the past decades, there has been great research performed in the area of the numerical symplectic integration of Hamiltonian systems (see [1–11]), the first-order Hamiltonian systems can be expressed as

(1) $$\begin{array}{c} \frac{dp_{i}}{dt}=-\frac{\partial H}{\partial q_{i}},\;\;\frac{dq_{i}}{dt}=\frac{\partial H}{\partial p_{i}},\;i=1,2,\ldots ,d, \end{array}$$

where *p*, *q* ∈ ℝ and *H* is a twice continuously differentiable function *H* : *U* → ℝ^2*d*^ (*U* ⊂ ℝ^2*d*^ is an open set). Hamiltonian systems often arise in different fields of applied sciences such as celestial mechanics, astrophysics, chemistry, electronics, and molecular dynamics (see [12]).

Quite often the solution of (1) exhibits an oscillatory character, so a numerical method which solved the Hamiltonian systems with oscillating solutions should be designed to pay attention to both the symplecticity and the oscillatory character. The phase-lag (or dispersion) property was introduced by Brusa and Nigro [13]. In the past few years, lots of work have been done in the construction symplectic methods for oscillating problems (see [6–8, 14–18]).

The general *s*-stage Runge-Kutta method is defined by

(2) $$\begin{array}{c} Y_{i}=y_{n}+h\sum_{j=1}^{s}a_{ij}f\left(x_{n}+c_{j}h,Y_{j}\right),\;i=1,2,\ldots ,s,\\ y_{n+1}=y_{n}+h\sum_{i=1}^{s}b_{i}f\left(x_{n}+c_{i}h,Y_{i}\right). \end{array}$$

**Lemma 1**

Assume that the coefficients of the method (2) satisfy the following relationship:

(3) $$\begin{array}{c} b_{i}a_{ij}+b_{j}a_{ji}-b_{i}b_{j}=0,\;1\leq i,\;\;j\leq s. \end{array}$$

Then the method is symplectic.

We consider constructing symplectic Runge-Kutta methods with high algebraic and dispersion order of the following format which is represented in a Butcher tableau, and the methods satisfy the condition (3) naturally,

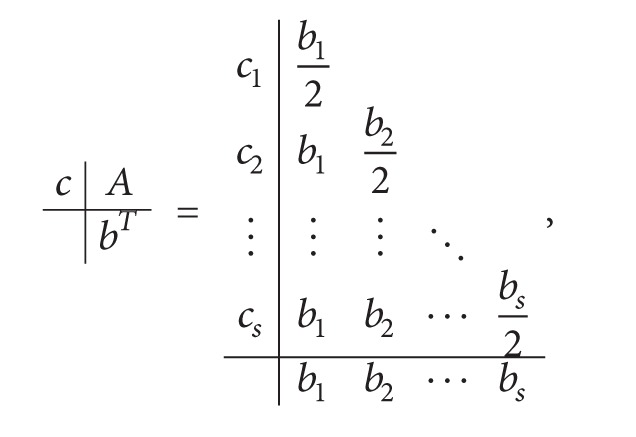
(4)
where *c*_*i*_ = ∑_*j*=1_^*s*^ *a*_*ij*_, *b*_*i*_ ≠ 0  (*i* = 1,2,…, *s*), *a*_*ij*_ = 0  (*i* < *j*).

The design and construction of numerical methods for Hamiltonian systems have been considered by several authors. In [3], a class of rational explicit symplectic integrators for one-dimensional oscillatory Hamiltonian problems is presented. In [4], Hairer and Wanner constructed symplectic Runge-Kutta methods using the W-transformation. In [6], Iserles constructed symplectic Runge-Kutta methods with real eigenvalues with the help of perturbed collocation. In [11], Sun gave a simple way to symplectic methods with the help of symplecticity conditions of partitioned Runge-Kutta methods. In [19], Sanz-Serna and Abia gave order conditions of symplectic Runge-Kutta methods.

The special symplectic methods (4) have been discussed by Suris [14], Qin and Zhang [10], Kalogiratou et al. [7, 8], Cong and Jiang [1], and Franco and Gómez [2]. In [14], method was derived with order *p* = 3. In [10], method was derived with order *p* = 4. In [2], five-stage symmetric method with algebraic order 4 and dispersion order 6 was presented. In [8], seven-stage method with algebraic order 5 dispersion order 6 and seven-stage method with algebraic order 4 dispersion order 8 were presented. In [1], method was derived with algebraic order 6.

In this paper, a nine-stage *A*-stable diagonally implicit symplectic Runge-Kutta (DISRK) method with algebraic order 6 and dispersion order 8 is constructed. The structure of the paper is as follows. In Section 2, we give some preliminary knowledge of dispersion of Runge-Kutta methods. In Section 3, nine-stage DISRK method with algebraic order 6 and dispersion order 8 is introduced. In Section 4, the stability and dispersive character of the proposed method are studied. In Section 5, numerical results are given to investigate the Hamiltonian quantity of the proposed method; the proposed method has been compared with the methods of Franco and Gómez [2], the methods of Kalogiratou et al. [8], and the method of Cong and Jiang [1], and they are all Runge-Kutta methods of the format (4); from the numerical experiments, the proposed method shows some superiority.

## 2. Preliminary Knowledge

The application of a Runge-Kutta method to the test problem

(5) $$\begin{array}{c} y^{\prime }=i\omega y,\;\omega \in R,\;i=\sqrt{1} \end{array}$$

leads to the numerical scheme

(6) $$\begin{array}{c} y_{n+1}=R\left(i\omega h_{n}\right)y_{n}, \end{array}$$

and *h*_*n*_ = *x*_*n*+1_ − *x*_*n*_, where the function *R*(*iv*) = *R*(*iωh*_*n*_) satisfies the relation

(7) $$\begin{array}{c} R\left(iv\right)=1+ivb{\left(I-ivA\right)}^{-1}e=\sum_{j=0}^{\infty }\beta_{j}{\left(iv\right)}^{j}, \end{array}$$

and for *j* ≥ 1, *β*_*j*_ = *bA* ^*j*−1^ *e*, *β*_0_ = 1, *e* = (1,1,…, 1)^*T*^, the numbers *β*_*j*_ depend only on the coefficients of the methods.

**Definition 2**

For a Runge-Kutta method the dispersion error (phase-lag error) and the dissipation error (amplification error) are given, respectively, by

(8) $$\begin{array}{c} \phi \left(v\right)=v-\arg\left(R\left(iv\right)\right),\\ d\left(v\right)=1-\left|R\left(iv\right)\right|. \end{array}$$

If *ϕ*(*v*) = *O*(*v* ^*q*+1^), then the Runge-Kutta method is said to have dispersion order *q*, and if *d*(*v*) = *O*(*v* ^*r*+1^), then the Runge-Kutta method is said to have dissipation order *r*. If at a point *v*, *d*(*v*) = 0, then the Runge-Kutta method has zero dissipation.

Moreover, if we consider the stability function

(9) $$\begin{array}{c} R\left(iv\right)=\beta_{0}+\beta_{1}v+\beta_{2}v^{2}+\cdots +\beta_{n}v^{n}+\cdots \end{array}$$

and collect the real and imaginary parts

(10) $$\begin{array}{c} R\left(iv\right)=A\left(v^{2}\right)+ivB\left(v^{2}\right), \end{array}$$

then the dispersion and dissipation errors can be written in the form

(11) $$\begin{array}{c} \phi \left(v\right)=v-\text{arc}\;\tan\left(v\frac{A\left(v^{2}\right)}{B\left(v^{2}\right)}\right),\\ d\left(v\right)=1-\sqrt{A^{2}\left(v^{2}\right)+v^{2}B^{2}\left(v^{2}\right)}. \end{array}$$

An alternative form for *R*(*z*)  (*z* = *iv*) is

(12) $$\begin{array}{c} R\left(z\right)=\frac{\det\left(I+z\left(eb^{T}-A\right)\right)}{\det\left(I-zA\right)}. \end{array}$$

For symplectic Runge-Kutta methods of format (12) always have

(13) $$\begin{array}{c} R\left(z\right)=\frac{\det\left(I+zA\right)}{\det\left(I-zA\right)}, \end{array}$$

hence they have |*R*(*z*)| = 1, so the method we discussed is zero dissipative method.

**Lemma 3 (see [20])**

A Runge-Kutta method is dispersive of order *q*, if the coefficients *β*_*j*_ in the *R*(*v*) satisfy the following conditions:

(14) β0j!−β1(j−1)!+β2(j−2)!−⋯+(−1)jβj=0, j=1,3,…,q−1,

and in addition *q* is even.

## 3. Construction of the New Method

Butcher proves that, if the stage number *s* and the coefficients  *a*_*ij*_, *b*_*i*_ are regarded as free parameters, then each equation of order conditions is independent of the others. However, as the Runge-Kutta methods (4), which satisfy the symplectic condition (3), the method coefficients are no longer free parameters and some order conditions turn out to be superfluous; Table 1 shows the number of order conditions of symplectic Runge-Kutta methods (SRK) and general Runge-Kutta methods (RK).

For the method of the format (4), the order conditions up to order 6 are (see [1])

(15) (1st) ∑ibi=1,  (3rd) ∑ibici2=13,(4rd) ∑ibici3=14,

(16) (5th) ∑ibici4=15,  ∑i,jbici2aijcj=110,    ∑i,j,kbiaijcjaikck=120,

(17) (6th) ∑ibici5=16,  ∑i,jbici2aijcj=112,∑i,j,kbiciaijcjaikck=124,  ∑i,j,kbici2aijajkck=136.

From Lemma 3, a SRK method of algebraic order *p* has at least dispersion order *p* + 1 if *p* is odd, *p* if *p* is even, so, a SRK method satisfying the above algebraic order conditions is the one with dispersion order 6, In order to achieve dispersion order 8; solving the dispersion condition (14), we get *β*_7_ = 1/7!; that is,

(18) $$\begin{array}{c} \sum_{i,j,k,l,m,n}b_{i}a_{ij}a_{jk}a_{kl}a_{lm}a_{mn}c_{n}=\frac{1}{7!}. \end{array}$$

Conditions (15), (16), (17), and (18) can be rewritten in the following form:

(19) τ1=∑ibi−1,  τ6=∑i,j,kbiaijcjaikck−120,τ2=∑ibici2−13,  τ7=∑ibici5−16,τ3=∑ibici3−14,  τ8=∑i,jbici2aijcj−112,τ4=∑ibici4−15,  τ9=∑i,j,kbiciaijcjaikck−124,τ5=∑i,jbici2aijcj−110,  τ10=∑i,j,kbici2aijajkck−136,τ11=∑i,j,k,l,m,nbiaijajkaklalmamncn−17!,T=(τ1,τ2,τ3,τ4,τ5,τ6,τ7,τ8,τ9,τ10,τ11).

To construct a nine-stage diagonally implicit symplectic Runge-Kutta method with algebraic order 6 and dispersion order 8, we only need to choose the free parameters *b*_*i*_  (*i* = 1,2,…, 9) to minimize the error norm,

(20) $$\begin{array}{c} A={||T||}_{2}. \end{array}$$

Minimizing the error norm, we have the DISRK methods parameters in Table 2 (M968: the first number denotes the number of stages, the second denotes the algebraic order, and the third denotes the dispersion order of the method).

## 4. Stability and Dispersive Error Analysis

In this section, we will investigate stability and dispersion character of the new method M968.

### 4.1. Stability

Considering a scalar test ordinary differential equation,

(21) y′=λy, λ∈C  Re(λ)<0.

Applying (2) to the test equation yields the stability difference equation of the form

(22) $$\begin{array}{c} y_{n+1}=R\left(z\right)y_{n},\;\;R\left(z\right)=1+zb^{T}{\left(I-zA\right)}^{-1}e, \end{array}$$

where *R*(*z*) is the stability function of the method and *I* is an identity matrix of size *s* × *s*, so *y*_*n*_ → 0 as *n* → *∞* if and only if |*R*(*z*)|<1, and the method is absolutely stable for those values of *z* for |*R*(*z*)|<1 holds. The stability region is defined as {*z* ∈ *C* : |*R*(*z*)|≤1}.

**Definition 4 (see [21])**

A Runge-Kutta method is said to be A-stable if its stability region contains *C* ^−1^, that is, the nonpositive half-plane {*z* | *Re*(*z*) < 0}.

For symplectic Runge-Kutta methods of format (4), we always have |*R*(*z*)| = 1. So we have that our new method M968 is *A*-stable.

**Figure 1:**
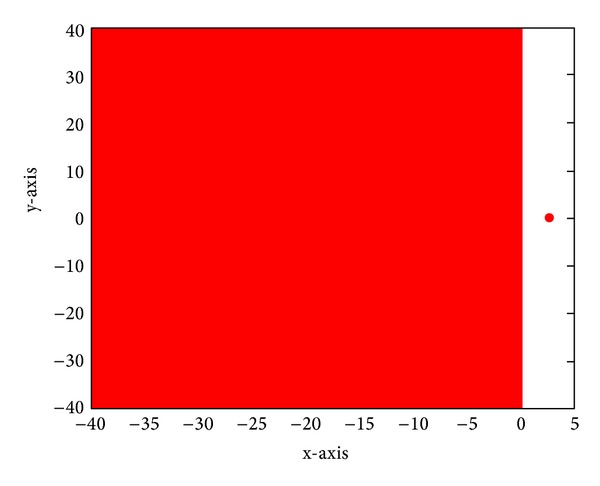
Stability region of the M968 method.

The stability region of the new method M968 is illustrated in Figure 1; from the figure, we can see that the points in the nonpositive half-plane and only few points in the right-plane satisfy |*R*(*z*)|≤1; that is, to say the new method M968 we discussed is *A*-stable method.

### 4.2. Dispersion Error

We compare the new method** M968** to some already known methods; the methods chosen to be tested are as follows.

1. Method** M546**: fourth-order symmetric DIRK methods for periodic stiff problems of Franco and Gómez (see [2]), a symmetric diagonally implicit Runge-Kutta method with five stages of algebraic order 4 dispersion order 6 was proposed.
2. Method** M756**: diagonally implicit symplectic Runge-Kutta methods with special properties of Kalogiratou et al. are a seven-stage method with algebraic order 5 and dispersion order 6 (see [8]).
3. Method** M748**: diagonally implicit symplectic Runge-Kutta methods with special properties of Kalogiratou et al. are a seven-stage method with algebraic order 4 and dispersion order 8 (see [8]).
4. Method** M766**: diagonally implicit symplectic Runge-Kutta methods of fifth- and sixth- order of Cong and Jiang is a seven-stage method with algebraic order 6 and dispersion order 6 (see [1]).
5. Method** M968** is proposed in the paper.

**Figure 2:**
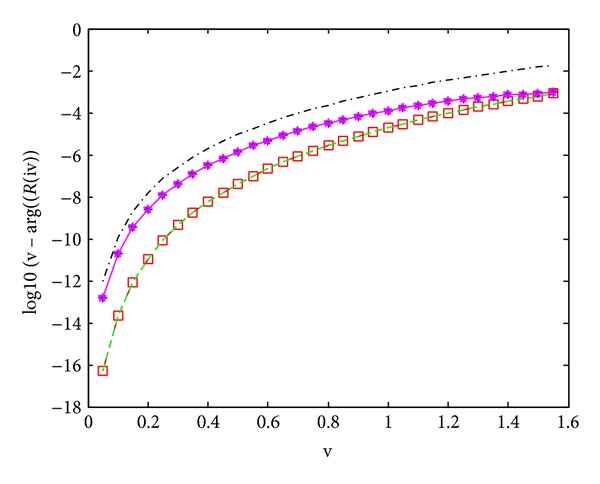
Difference in dispersion. Methods used: (i)–□–., in red, our method, M968. (ii)–, in green, M748 ([8]). (iii)–∗–, in blue, M766 ([1]). (iv)–., in black, M546 ([2]). (v)–*⋆*–, in pink, M756 ([8]).

Figure 2 shows the dispersion error of the five compared methods,; from the figures, we see that the dispersive error curve of M968 and M748 appears to overlap, for they have the same dispersive order, and this is the case for M766 and M756. On the other hand, we can see the dispersion orders of the M968 and M748 are the highest ones in the compared five methods; the lowest one is the method M546 of Franco and Gómez ([2]).

## 5. Numerical Experiments

### 5.1. Numerical Examples

In this numerical study, we are interested in the errors of the Hamiltonian quantity. Three well known Hamiltonian problems from the literature were chosen for our test.

#### 5.1.1. Harmonic Oscillatory System

Consider

(23) $$\begin{array}{c} q^{\prime }=p,\;\;p^{\prime }=-q. \end{array}$$

The Hamiltonian function is

(24) $$\begin{array}{c} H=\frac{1}{2}\left(p^{2}+q^{2}\right). \end{array}$$

The exact solution is

(25) $$\begin{array}{c} \left(\begin{array}{c} p\left(t\right)\\ q\left(t\right) \end{array}\right)=\left(\begin{array}{cc} \cos\left(t\right) & -\sin\left(t\right)\\ \sin\left(t\right) & \cos\left(t\right) \end{array}\right)\left(\begin{array}{c} p\left(0\right)\\ q\left(0\right) \end{array}\right), \end{array}$$

where *p*(0) = −0.1, *q*(0) = 0.3.

**Figure 3:**
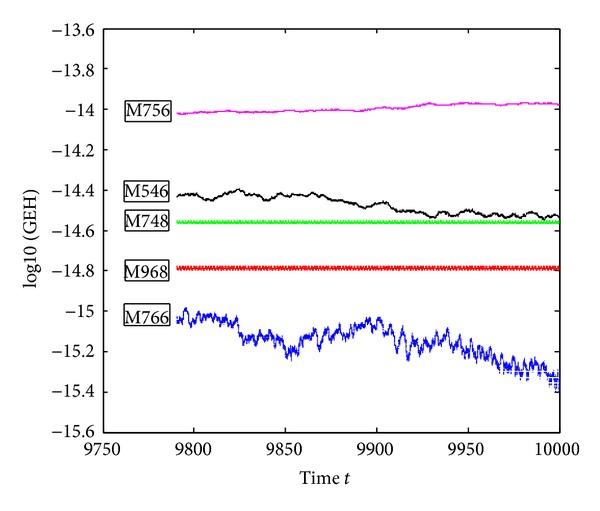
Errors of the Hamiltonian function of (23) on [0,10000], with *h* = *π*/300.

We get the Hamiltonian error GEH = ||*H*_*n*_ − *H*_0_|| of the compared five methods on the interval *t* ∈ [0,10000] and the step-size *h* = *π*/300.  Figure 3 shows the last 20000-step Hamiltonian quantity error. From the figure, we can see that the accuracy of M968 is slightly inferior to the M766 and more better than M748, M756, and M546. The M766 is of more accuracy than the M968, for it has lower computational cost than M968 when solving problem 1, but on the other hand, the Hamiltonian quantity error of the M766 ranges from 10^−15^ to 10^−15.4^ in the last 20000 steps; it does not keep the Hamiltonian quantity unchanged and the same case for M546 when the step-size *h* = *π*/300.

#### 5.1.2. The Mathematical Pendulum

It is a famous model of nonlinear differential equations in classical mechanics that can be written as

(26) $$\begin{array}{c} p^{\prime }=-\sin q,\;\;q^{\prime }=p. \end{array}$$

The Hamiltonian function is

(27) $$\begin{array}{c} H=\frac{1}{2}p^{2}-\cos q. \end{array}$$

The initial values are *p*(0) = 0, *q*(0) = 0.5.

**Figure 4:**
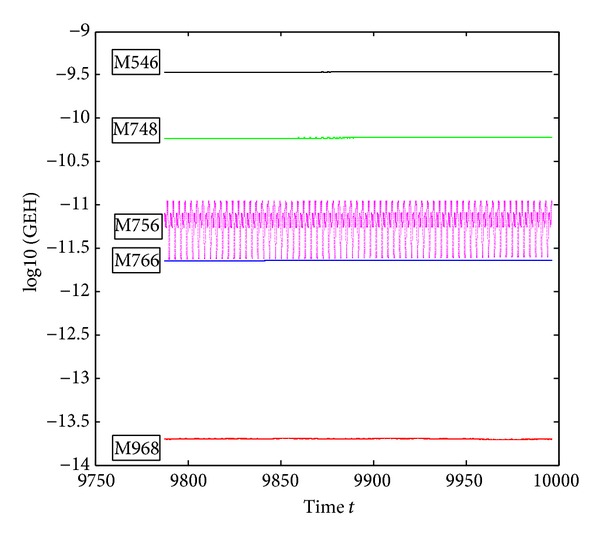
Errors of the Hamiltonian function of (26) on [0,10000], with *h* = *π*/300.

We get the Hamiltonian error GEH = ||*H*_*n*_ − *H*_0_|| of the compared five methods on the interval *t* ∈ [0,10000] and the step-size *h* = *π*/300; Figure 4 shows the last 20000 steps of Hamiltonian quantity error. From the figure, we can see that the M968 is the best one in the five compared methods, the Hamiltonian error of M756 mainly ranges from 10^−11.6^ to 10^−10.8^; the others can keep the Hamiltonian quantity unchanged in the last 20000 steps.

#### 5.1.3. The Two-Body Problem

Consider

(28) p1′ =−q1(q12+q22)3/2,  p2′=−q2(q12+q22)3/2,p1′ =p1,  p2′=p2.

The Hamiltonian function is

(29) $$\begin{array}{c} H\left(p,q\right)=\frac{1}{2}\left(p_{1}^{2}+p_{2}^{2}\right)-\frac{1}{{\left(q_{1}^{2}+q_{2}^{2}\right)}^{1/2}}, \end{array}$$

where *p* = (*p*_1_, *p*_2_)^*T*^ and *q* = (*q*_1_, *q*_2_)^*T*^ are the velocity and position vectors, with the initial conditions

(30) $$\begin{array}{c} p_{1}\left(0\right)=0,\;\;p_{2}\left(0\right)=1,\\ q_{1}\left(0\right)=1,\;\;q_{2}\left(0\right)=0. \end{array}$$

The exact solution of this initial value problem is given by

(31) $$\begin{array}{c} p_{1}\left(t\right)=-\sin\left(t\right),\;\;p_{2}\left(t\right)=\cos\left(t\right),\\ q_{1}\left(t\right)=\cos\left(t\right),\;\;q_{2}\left(t\right)=\sin\left(t\right). \end{array}$$

The system has the energy *H* = (1/2)(*p*_1_(*t*)^2^ + *p*_2_(*t*)^2^)−(1/(*q*_1_(*t*)^2^ + *q*_2_(*t*)^2^)^1/2^) and the angular momentum *M* = *q*_1_(*t*)*p*_2_(*t*) − *q*_2_(*t*)*p*_1_(*t*) as conserved quantities.

**Figure 5:**
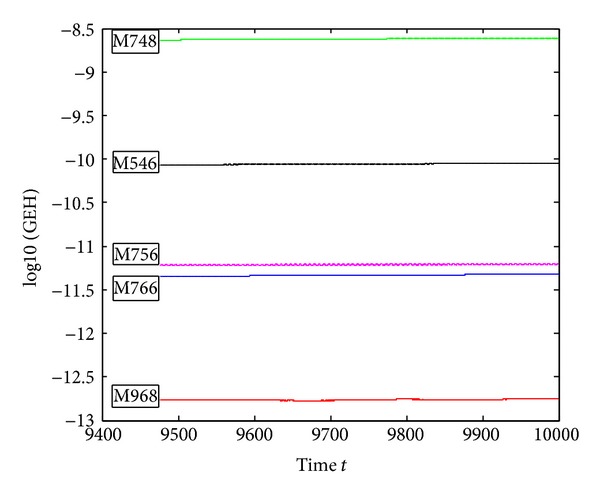
Errors of the Hamiltonian function of (28) on [0,10000], with *h* = *π*/60.

**Figure 6:**
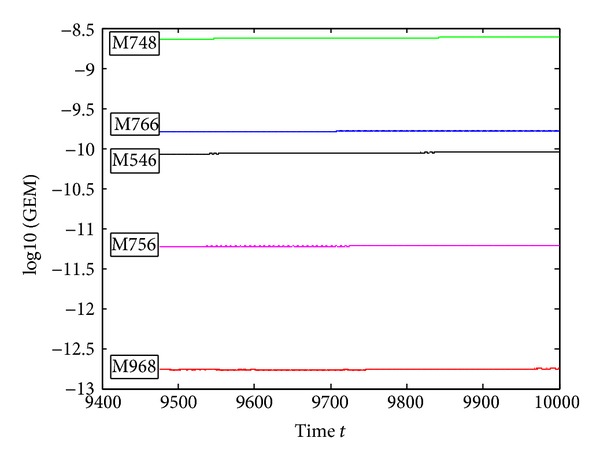
Errors of the Momentum function of (28) on [0,10000], with *h* = *π*/60.

We check the preservation of the Hamiltonian *H* and the angular momentum *M* of the compared five methods when solving the two-body problem. The last 10000-step global Hamiltonian error GEH = ||*H*_*n*_ − *H*_0_|| and the global angular momentum error GEM = ||*M*_*n*_ − *M*_0_|| were plotted in Figures 5 and 6 with the interval *t* ∈ [0,10000] and the step-size *h* = *π*/60, where *H*_*n*_ and *M*_*n*_ are the computed values of *H* and *M* at each integration point *t*_*n*_. From the figures, we can see that the M968 is the best one in the five compared methods.

## 6. Conclusion

Here we have constructed a diagonally implicit symplectic nine-stage Runge-Kutta method with algebraic order 6 and dispersion order 8. As we can see from the stability region and difference in dispersion, the new method is *A*-stable method and more easily implemented than general fully implicit methods. The numerical experiments carried out with some oscillatory Hamiltonian systems show that the new method is as competitive as the existing Runge-Kutta methods of the same type.

## Figures and Tables

**Table 1:** Number of order conditions for RK and SRK to order 6.

Order	RK Method	SRK Method
1	1	1
2	2	1
3	4	2
4	8	3
5	17	6
6	37	10

**Table 2:** The value of *b*_*i*_ (*i* = 1,2,…, 9) and *A*.

*b*_1_	*b*_2_	*b*_3_	*b*_4_	*b*_5_
2.44398640327406	−2.46929010453909	0.28158632623993	0.50745789725108	1.17888214306555

*b*_6_	*b*_7_	*b*_8_	*b*_9_	*A*

−2.31558614555863	2.35136242638295	−1.24653876689005	0.26813982077420	3.496139974472668*e* − 014
